# An Evaluation System for the Contact Electrification of a Single Microparticle Using Microelectromechanical-Based Actuated Tweezers

**DOI:** 10.3390/s18061835

**Published:** 2018-06-05

**Authors:** Daichi Yamaguchi

**Affiliations:** RICOH Company, Ltd., 810 Shimo-Imaizumi, Ebina-shi, Kanagawa 243-040, Japan; daichi.yamaguchi@jp.ricoh.com; Tel.: +81-50-3814-3081

**Keywords:** cantilever, nanotweezers, manipulation, contact electrification, single particle

## Abstract

The image quality of laser and multi-function printers that make use of electrophotography depends on the amount of surface charge generated by contact electrification on the toner particles. However, because it has been impossible to experimentally evaluate such amounts under controlled contact conditions using macroscopic measurements, theoretical elucidation of the contact electrification mechanism has not progressed sufficiently. In the present study, we have developed a system to experimentally evaluate the contact electrification of a single particle using atomic force microscopy (AFM) and nanotweezers (microelectromechanical systems (MEMS)-based actuated tweezers). This system performs, in succession, (i) a contact test that makes use of the nanotweezers and three piezoelectric stages, and (ii) an image force measurement using the AFM cantilever. Using this system, contact electrification was evaluated under controlled conditions, such as the contact number and the indentation depth. In addition, differences in contact electrification due to the amount of external surface additives were investigated. The results reveal that a coating with external additives leads to a decrease in the amount of contact electrification due to a reduction in the contact area with the substrate.

## 1. Introduction

Electrophotography-based laser and multi-function printers have primarily been used for general document printing in offices. In addition to office use, their application is expanding to production printing, e.g., utilization by in-house printing departments of large enterprises and small-lot printing in printing shops, as the image quality of these printers has improved. However, it is still highly desirable to improve their performance in terms of printing speed and image.

In electrophotography, contact-electrified toner particles are moved by an electric field to form an image on paper [1]. Because the amount of surface charge on the toner particles significantly affects the printed image quality, it is critical to understand contact electrification (also known as contact charging or tribocharging). Despite being a well-known phenomenon [2], contact electrification is not predictable quantitatively and does not have an established theory, primarily because evaluation under a controlled state of contact is difficult. The strength and polarity of the contact charges depend on factors such as the surface materials, surface roughness (which determines the contact area), load, and contact time, which for the most part are non-uniform on the microscopic scale. Since the frequency and intensity of contact are influenced by powder flowability, especially in the contact electrification of powders, the phenomenon is more complicated. As a result, it has been difficult to accumulate reproducible data and carry out measurements under controlled conditions.

Atomic force microscopy (AFM), a powerful tool for visualizing a microscopic state based on the force measured between a sample and the tip of the AFM cantilever, has been utilized for the study of controllable contact electrification in a reproducible manner. Charged states can also be visualized using electrostatic force microscopy (EFM) or the Kelvin probe force microscopy (KFM) method [3]. Additionally, manipulation of the cantilever, such as approach/withdraw cycling or scanning, is also possible with AFM. By combining the above two functions, Terris et al., Morita et al., and Sun et al. generated charge through contact between an AFM tip and a substrate, and observed the charged state using AFM [4,5,6]. Although Lowell et al. reported an evaluation of the contact electrification between a milli-sphere of metal and resins under controlled conditions [7], the above studies enabled such controlled evaluations microscopically.

Terris et al. observed contact charging between a Ni tip and PMMA, and reported that the charged region was much larger than the expected contact area. Morita et al. observed the electric charge generated by bringing a voltage-applied conductive cantilever into contact with a SiO_2_ substrate. Sun et al. reported that the charge generated by friction between a cantilever and SiO_2_ substrates under different load conditions can be observed using AFM. Although these studies were advanced in that charged states could be reproducibly generated and evaluated, the evaluation was limited to contact charging between the tip material of the cantilever and the sample. In other words, they did not conduct any particle (powder) contact charging studies.

Colloidal probe atomic force microscopy (CP-AFM) makes full use of the reproducibility of AFM and the ability to study contact charging of particles. In CP-AFM, a single particle is glued to the tip of the cantilever, and the interaction between the particle and a substrate can be investigated [8,9]. Specifically, CP-AFM can be used to investigate contact electrification under controlled contact conditions, including the contact number, contact time, and contact load. The electrostatic force on the particle can be measured over a long range as a force-displacement curve. Some groups have made use of these advantages of CP-AFM to measure the contact electrification of particles. For example, Gady et al. reported that polystyrene particles are charged upon contact with highly oriented pyrolytic graphite, but not gold [10]. Eve et al. reported that salbutamol particles become charged by repeated contact with PTFE [11]. Bunker et al. reported that the scanning of lactose particles on glass generates more charge than repeated local contacts [12].

However, CP-AFM has considerable limitations, e.g., a particle must be glued to the AFM cantilever with epoxy resin, which is very time-consuming. Depending on the equipment and skill of the researcher, it can take several hours to fix a particle to the cantilever, allow the epoxy resin to dry, and perform an AFM measurement. Moreover, the number of measurements that can be performed is limited. Therefore, it is difficult to evaluate the variation among particles in terms of, e.g., surface roughness, diameter, and material complexity. As a result, such investigations are impractical and have not expanded beyond fundamental research.

However, there are some reports on the study of single-particle contact electrification under controlled conditions. Watanabe et al. measured charge generation due to a single impact between a particle and a target plane, and studied the relationship between the generated charge and the impact velocity [13]. Although this method enables the charging of the particles to be investigated under a controlled state, the amount of charge generated by only one contact event can be studied. It is difficult to simulate realistic contact conditions, such as random contact positions on the particle surface and friction. Moreover, Park et al. evaluated contact charging by manipulating particles with an optical trap [14]. This method can potentially be used to study the charging of particles under various contact conditions. However, optical traps have significant limitations for practical contact charging studies. For example, light-sensitive materials cannot be evaluated as powders and substrates because of material deterioration due to the laser irradiation [15]. In addition, because the force acting on an object typically lies in the range of 1 to 100 pN [16], which is much smaller than the typical adhesive force on a microparticle of a few to hundreds of nN, vibration of the substrate to decrease the adhesion between the particle and the substrate is required [16]. Although this is an advanced method, stable evaluation is limited to relatively low-adhesive particles and substrates. It is also difficult to carry out evaluations to raise the contact load on particles. It should be noted that the original usage of optical trapping is mostly limited to trapping objects floating in liquid.

In this paper, we present a unique method for evaluating the contact electrification of a single particle using nanotweezers (microelectromechanical systems (MEMS)-based actuated tweezers) and an AFM cantilever. We have previously proposed a technique that allows for much faster measurement of the charge on a single toner particle than CP-AFM [17,18]. In the previous report, we showed that the charge obtained by our method is linearly correlated with that obtained using the conventional blow-off method. The present approach combines this method with manipulation by nanotweezers, enabling the simulation of contact electrification of a single particle under controlled contact conditions. Because the particle is manipulated through mechanical gripping, the various materials can be studied and experimental contact conditions, including friction, can be applied. The details of the experimental setup are first described, after which differences in the triboelectric characteristics of various powders are discussed.

## 2. Materials and Methods

**Figure 1:**
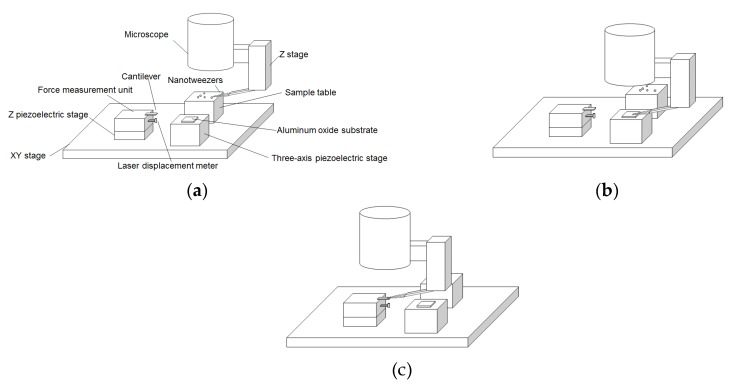
Experimental apparatus for evaluating the contact electrification of a single particle: (**a**) Picking up a toner particle; (**b**) Contact test; (**c**) Image force measurement.

Figure 1 shows the system for evaluating single-particle contact electrification. The technique involves picking up a particle, conducting a contact test between the particle and a substrate, measuring the image force of the particle, and calculating the charge from the measured image force. This system consists of nanotweezers with a proximity sensor (Aoi Electronics Co., Ltd., Kagawa, Japan), a three-axis piezoelectric stage (stage: SFS-H60XYZ(CL), controller: FINE-503(CL); Sigmakoki Co., Ltd., Saitama, Japan) used for the contact test, a force measurement unit with a cantilever, an optical microscope, and two translation stages (an XY stage and a Z stage). It differs compared with the previous work [18] in that a three-axis piezoelectric stage is now implemented in the contact test. The transitions among each process were carried out by moving the XY stage.

### 2.1. Picking up a Single Particle

**Figure 2:**
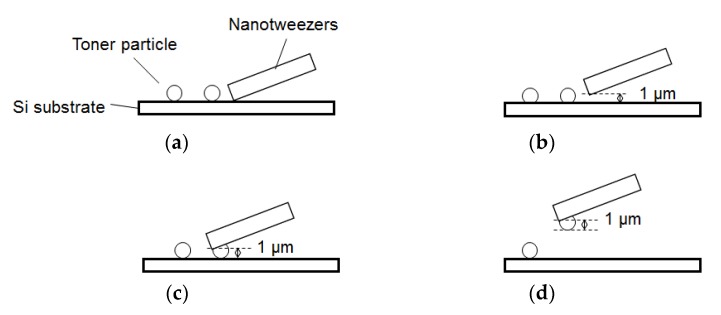
Procedure for picking up a particle: (**a**) Form contact between the nanotweezers and the Si substrate; (**b**) Move the nanotweezers upward by 1 μm; (**c**) Move nanotweezers horizontally and grip the particle; (**d**) Pick up the particle.

In the proposed system, the nanotweezers, which are made of silicon, are used to pick up a single toner particle. Before picking up a particle, the nanotweezers are brought into contact with the substrate near the particle and moved upward by 1 μm as shown in Figure 2. This ensures that the bottom of the particle, not the nanotweezers, touches either the substrate for the contact test or the cantilever. Contact detection between the nanotweezers and the substrate is carried out by the proximity sensor of the nanotweezers, which determines the contact with an object based on the oscillation of the arm in the direction parallel to the substrate [17]. The positional relationship between the nanotweezers and the particle is adjusted using the XY stage according to the optical microscope image as shown in Figure 2a.

### 2.2. Contact Test

**Figure 3:**
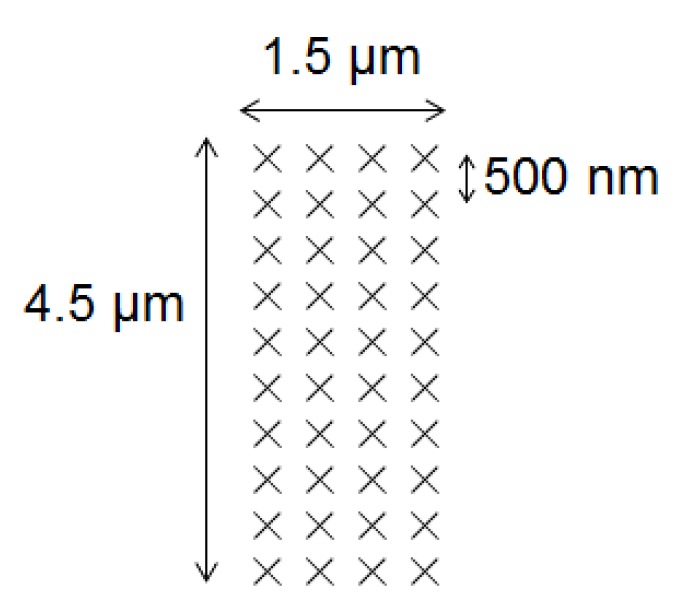
Contact positions on the aluminum oxide substrate.

**Figure 4:**
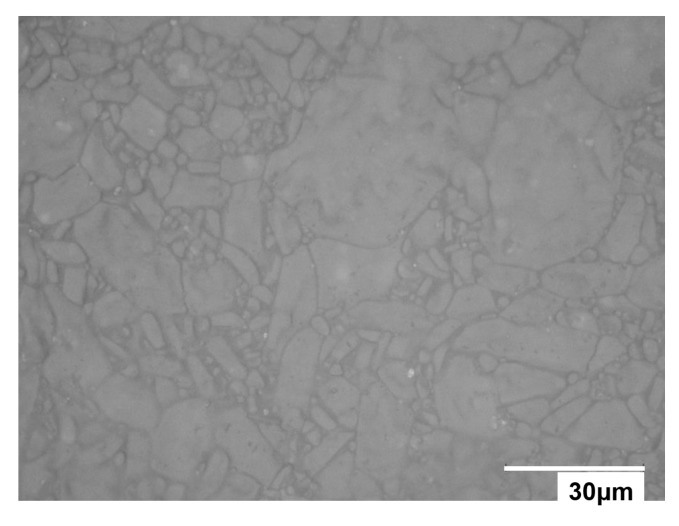
Optical micrograph of the aluminum oxide substrate.

The contact tests between the particle and the substrate were carried out by bringing the toner into contact with the substrate at 40 positions arranged with a 500-nm pitch as shown in Figure 3. Since toners that tend to become negatively charged were used in the present study, as described in Section 2.5, aluminum oxide, which has a tendency to become positively charged [2,19,20], was chosen as a suitable substrate. A plate of aluminum oxide with a thickness of 1 mm (AL-017518, The Nilaco Corporation, Tokyo, Japan) was prepared as the contact substrate, for which an optical micrograph is shown in Figure 4.

**Figure 5:**
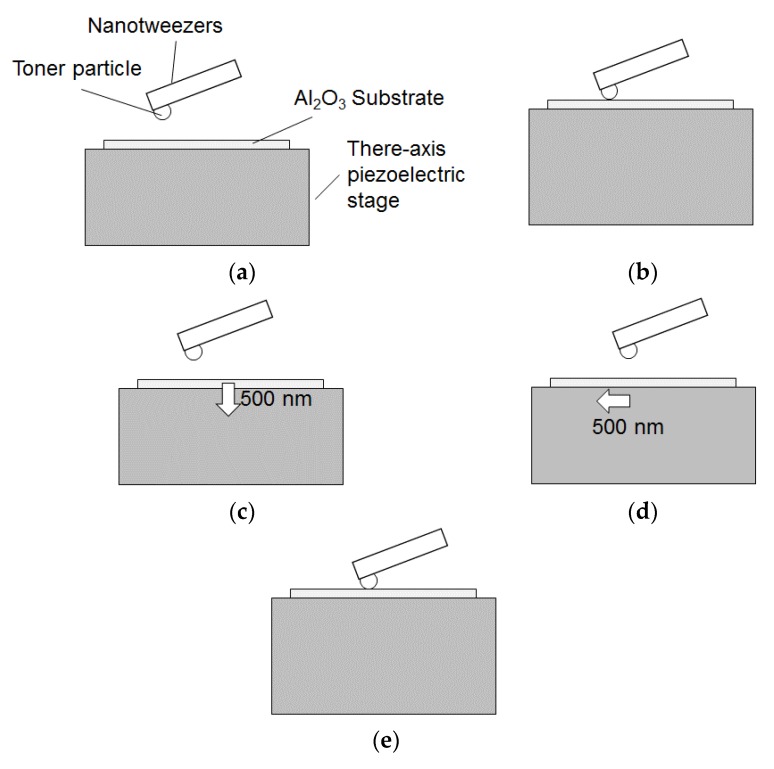
Procedure for a contact test between a toner particle and an aluminum oxide substrate: (**a**) before approach; (**b**) approach/contact particle; (**c**) withdraw from particle; (**d**) horizontal translation; (**e**) approach/contact particle.

The contact test starts with the upward approach of the aluminum oxide substrate in the Z direction toward the toner as shown in Figure 5. After contact is made, the three-axis piezoelectric stage moves the substrate downward by 500 nm, re-separating the toner from the substrate, and moves to the next contact position in the X or Y direction. This process of approach/contact, withdraw, and horizontal movement is carried out a total of 40 times.

The contact between the toner and the substrate is detected by the proximity sensor of the nanotweezers. We verified that this contact detection method works when the nanotweezers are gripping a particle, and provide the details of the verification in Appendix A.

### 2.3. Image Force Measurement

After the contact test, the XY stage slides the force measurement unit below the nanotweezers, which are gripping the particle. Although the system used for the image force measurement has been previously described in detail [18], here it differs in that the AFM cantilever was milled using a focused ion beam (FIB) to improve the measurement sensitivity by reducing the spring constant. In the contact test, the charged area is limited to around the bottom of the particle. In contrast, here the entire surface of the particle is charged through powder mixing and stirring. Thus, during image force measurement, the deflection of the cantilever must be made as high as possible to maximize the measurement sensitivity.

**Figure 6:**
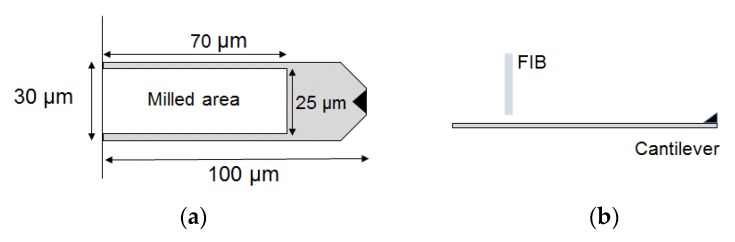
Schematic diagram of focused ion beam (FIB) processing of the cantilever: (**a**) Dimensions of the processing area; (**b**) positional relationship between the FIB and the cantilever.

The gold-coated cantilever (BL-RC150VB; resonance frequency: 13 kHz, spring constant: 0.006 N/m; Olympus, Tokyo, Japan), which at present has the lowest commercially available spring constant, was processed using a FIB-SEM system (NVision 40; Carl Zeiss, Jena, Germany). The original size of the cantilever was 100 μm in length, 30 μm in width, and 180 nm in thickness. We milled a rectangle of 70 μm in length and 25 μm in width from the base to leave intact the area where the laser (spot size: ~20 μm) is reflected as shown in Figure 6a. The processing was carried out with an ion voltage of 30 kV and a current of 6.5 nA. The Ga ion beam irradiated the tip side of the cantilever as shown in Figure 6b.

The FIB-processed cantilever is mounted on a uniquely designed holder, and a laser displacement meter (SI-F01; resolution: 1 nm; Wavelength of light source: 820 nm; Keyence, Osaka, Japan) monitors the cantilever deflection. The distance between the cantilever and the sensor head of the laser displacement meter is approximately 100 μm, meaning that the intensity of the light reflected from the cantilever is sufficient for the displacement measurement. The cantilever is grounded. During the image force measurement, the voltage on the cantilever is set to zero. The cantilever is fixed such that the tip faces the laser displacement meter, because the flat side is used to make contact with the particle gripped by the nanotweezers. A piezoelectric stage (PI-Japan Co., Ltd., Tokyo, Japan; PIHera Precision Z-Stage P-621.ZCD) moves the cantilever holder upward at a velocity of 10 μm/s until contact is made with the toner particle. If the toner particle is charged, the cantilever is attracted to it by the image force. By determining the deflection of the cantilever and the displacement of the piezoelectric stage, the deflection-displacement curve can be obtained. The sampling time of the deflection of the cantilever is 200 μs. Because the cantilever moves together with the laser displacement meter, the cantilever deflection is changed by the force acting on the cantilever, not by the motion of the piezoelectric stage.

In the present study, we present cantilever deflection-displacement curves rather than force-displacement curves to demonstrate the effect of the FIB processing of the cantilever. The image force can be calculated by multiplying the measured deflection by the spring constant of the cantilever.

### 2.4. Charge Calculation

The amount of charge generated during the contact test can be calculated from the image force, which is obtained by multiplying the deflection and the spring constant of the cantilever. The measured force-displacement curve is fitted to the image force equation using the method of least squares. In the calculation, a parameter called the imaginary center of charge is introduced to model the non-uniform charge distribution on a single particle as an equivalent point charge. The details of the calculation have been described in a previous report [18].

### 2.5. Material Preparation

Two kinds of electrophotographic toners with a tendency to charge negatively were prepared. One was manufactured using a pulverizing method to provide an irregular particle shape for verifying the sensitivity of the FIB-processed AFM cantilever. The other toner was also manufactured using a pulverizing method, but was heat treated to provide spherical particles, which simplifies the interpretation of the contact test results. The toner particles had a number average diameter of approximately 5 μm. Both toners were treated with silica external additives in the following amounts: 0, 0.3, or 1.3 wt % for spherical particles and 2.0 wt % for irregular-shaped particles. The additives are hydrophobic and have an average particle diameter of around 10 nm. To verify the sensitivity of the cantilever, the charge on the toners was adjusted by mixing the toners with carriers produced under different coating conditions, and the charge-to-mass ratios were obtained using the blow-off method [18]. The carrier is a powder used in electrophotography, with larger particles than toner and tends to be charged with polarity opposite that of toner [21]. The particles were deposited in isolation on the Si substrate. A toner mixed without carrier particles, which was estimated to be substantially uncharged, was also prepared to verify the sensitivity of the cantilever and the contact test.

## 3. Results and Discussion

### 3.1. Evaluation of FIB-Processed Cantilever

**Figure 7:**
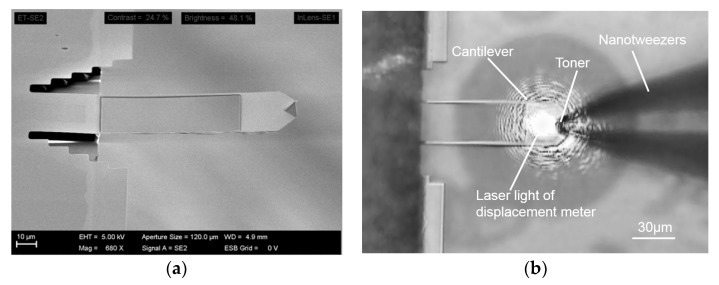
Micrographs of the FIB-processed cantilever: (**a**) Scanning electron microscopy image of the FIB-processed cantilever; (**b**) Optical microscopy image of the FIB-processed cantilever mounted on the piezoelectric stage and nanotweezers gripping the toner particle.

A scanning electron microscopy image of the FIB-processed cantilever is shown in Figure 7a. The image confirms that the FIB milling was performed without a large deformation and according to the intended dimensions shown in Figure 6. The FIB-processed cantilever mounted on the piezoelectric stage along with the laser spot is shown in Figure 7b. The laser beam is seen to be fully incident on the cantilever.

**Figure 8:**
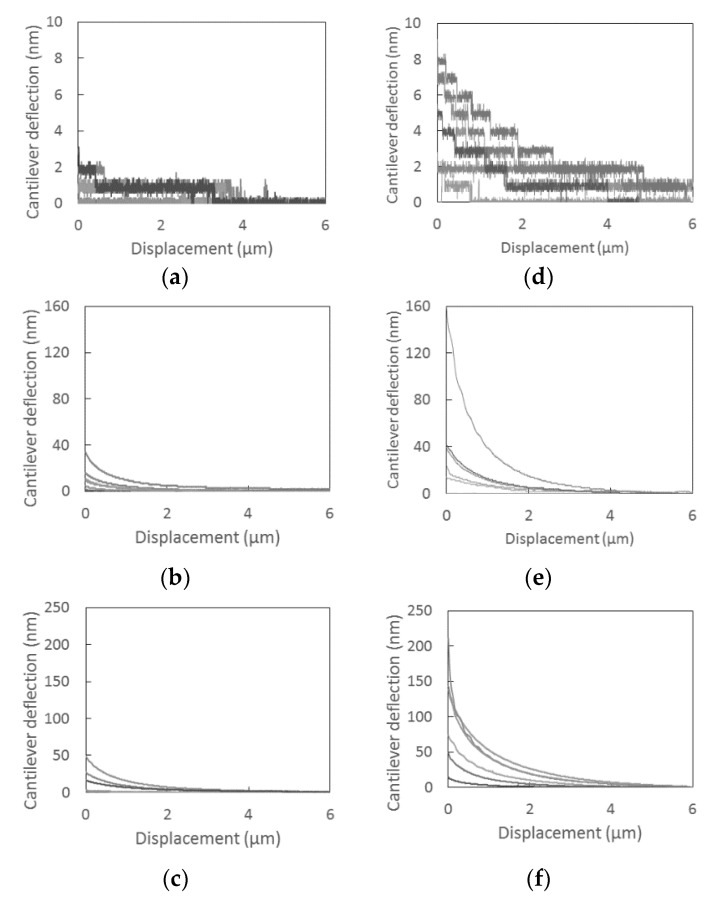
Deflection-displacement curves for (**a**–**c**) non-processed and (**d**–**f**) FIB-processed cantilevers, and charge-to-mass-ratios of (**a**,**d**) 0 μC/g (not mixed with carrier); (**b**,**e**) q/m = −13.6 μC/g; (**c**,**f**) q/m = −20.7 μC/g. The charge-to-mass ratios q/m were obtained using the blow-off method. Note that the toner particles were charged by mixing with the carrier, not contact-electrified. Also note that the Y-axis range is different among each charge-to-mass ratio to more easily compare the effect of FIB-milling.

Figure 8 shows the cantilever deflection-displacement curves measured for non-processed and FIB-processed cantilevers and samples with a charge-to-mass ratio of 0, −13.6, or −20.7 μC/g. It should be noted that the toner particles were charged by mixing with the carrier, not contact-electrified using the proposed system. Each plot includes the results for six particles selected randomly from a sample of a given charge-to-mass ratio. The variation in the image force among the five randomly selected particles is due to variations such as size, shape, and surface composition. Figure 8 demonstrates that the FIB processing increases the deflection of the cantilever due to the increase in the image force, which leads to an improvement in the charge measurement sensitivity of the proposed system.

The spring constant of a cantilever is proportional to the width of the cantilever [22]. The width of the non-processed cantilever is 30 μm and the width of the milled area is 25 μm. Therefore, the sum of the widths on both sides of the FIB-processed cantilever is approximately 5 μm, which is one-sixth that of the non-processed cantilever. Based on the manufacturer-specified value of 0.006 N/m for the non-processed cantilever, the spring constant for the FIB-processed cantilever is estimated to be 0.001 N/m.

### 3.2. Effect of External Additives on Contact Electrification

**Figure 9:**
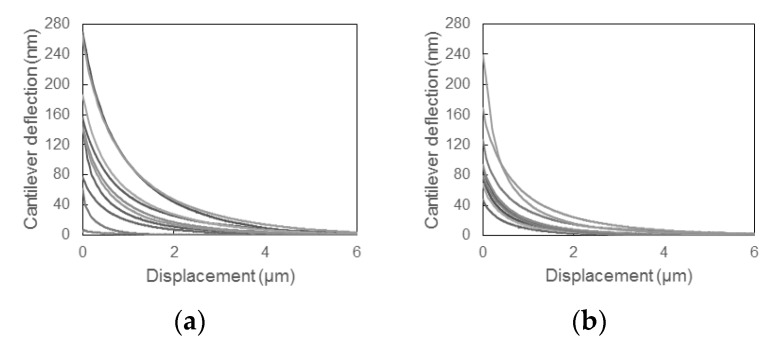

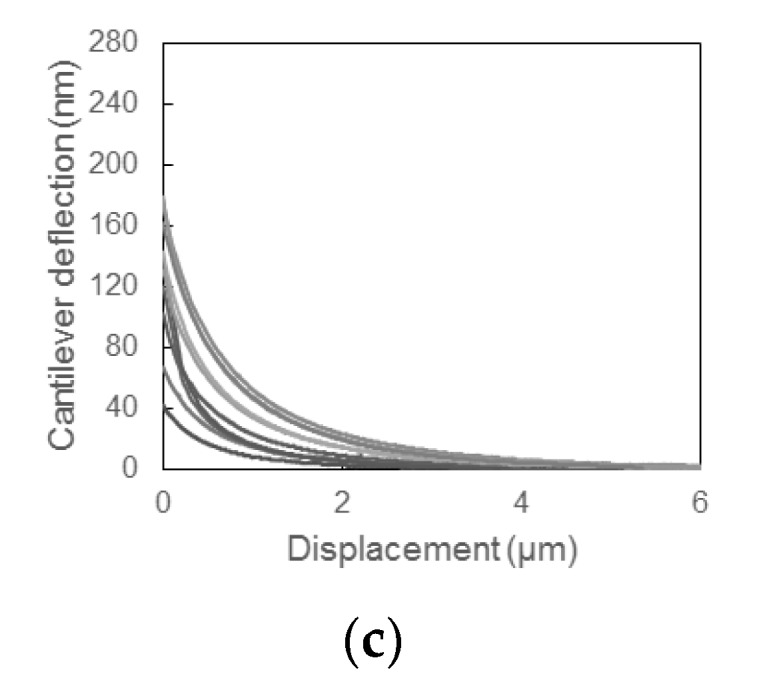
Cantilever deflection-displacement curves for contact-electrified particles treated with different amounts of external additives: (**a**) 0 wt %; (**b**) 0.3 wt %; (**c**) 1.3 wt %.

Figure 9 shows deflection-displacement curves for particles treated with 0, 0.3, or 1.3 wt % external additives and contact-electrified using the procedure described in Section 2.1. Each graph includes the results for 10 particles selected randomly from each of the three samples. The changes in deflection due to long-range attractive forces are observed clearly. This demonstrates that the electrostatic charge on the toner particle is generated during the contact test. Sufficient cantilever deflection is observed in all plots, which is the effect of reducing the spring constant by FIB processing.

**Figure 10:**
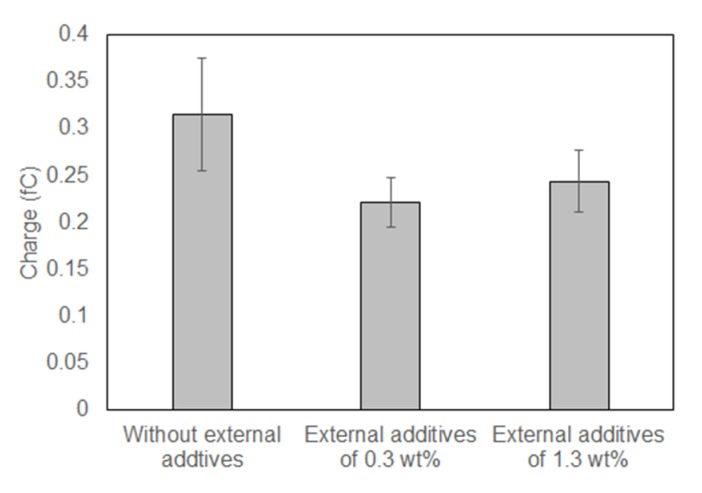
Charge calculation results for contact-electrified particles treated with different amounts of external additives. Error bars represent standard error.

Figure 10 shows the average charge calculated from Figure 9. The calculations were carried out using a spring constant of 0.001 N/m based on the discussion in Section 3.1. The calculated charges lie in the range of 0.2–0.4 fC. The charge of a single toner particle is estimated to be several fC from the typical charge-to-mass ratio of tens of μC/g. Moreover, we have shown that the charge obtained using our method lies in the range of 0.2–0.8 fC when the particles are charged by mixing with the carrier used in the previous work [18]. It is expected that the entire surfaces of the particles are charged under the above conditions. In Figure 9, the particles are partly charged, and the charge is estimated to be a fraction of that of an entirely charged particle. The obtained value is considered to be reasonable.

**Figure 11:**
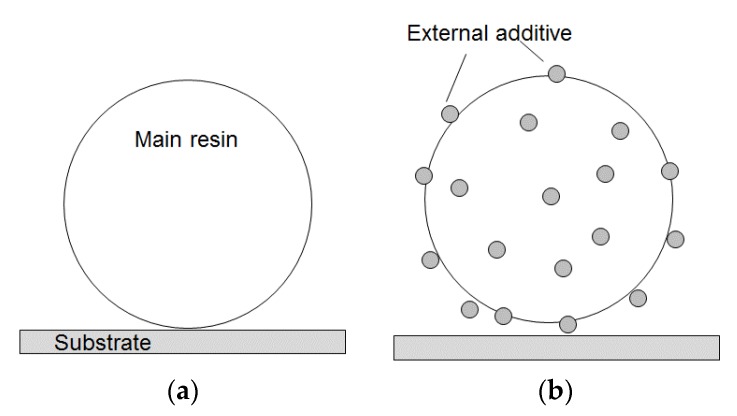
Effect of external additives on contact between a toner particle and a substrate: (**a**) without external additives; (**b**) with external additives.

Figure 9 indicates that a coating with external additives leads to a decrease in the amount of charge induced through contact with the aluminum oxide substrate. It is well-known that coating particle surfaces with external additives leads to a decrease in the particle–substrate contact area because additives act as a spacer as illustrated in Figure 11 [23]. Figure 10 shows that a decrease in the contact area results in a decreased amount of generated charge. However, an increase in the amount of silica additives does not cause a monotonic decrease in the amount of charge accumulated during the contact electrification. As an external additive, silica is strongly negatively charged [2,19,20]. An increase in the amount of additive seems to facilitate negative charging of the toner particles, which compensates for the reduction in the particle–substrate contact area.

It should be noted that the data in Figure 9 was obtained using only one cantilever. Thirty cantilevers would be required to obtain the same results as those in Figure 9 using CP-AFM. It is time-consuming to prepare a large number of colloidal probes, and the cost and time required are expected to be several tens of times higher than those required for the proposed method. In addition, the high throughput enables the investigation of various contact electrification characteristics, such as the material and the surface roughness of the powder. Specifically, the material and surface roughness of the particles change the contact frequency of the powder (flowability) and the propensity for contact electrification (tendency to gain or lose electrons). Macroscopic measurements of the amount of charge after mixing and stirring cannot evaluate these contributions independently. The contact electrification mechanism is more complicated for composite materials with irregular shapes or external additive coatings. The controlled conditions—number of contacts and loading—provided by the proposed system allow us to investigate such a complex mechanism.

The proposed method has the remaining issue that its quantitativeness has not been sufficiently verified, although it enables qualitative comparison among particles. The spring constant of the cantilever tends to be inherently uneven due to the difficulty of controlling the thickness of the cantilevers. Moreover, it may deviate from the current calculated value (0.001 N/m) because the FIB-milling area is not exact as shown in Figure 7a. Langlois et al. reported that the calibrated results differed from the nominal values by up to 300% [24]. The amount of charge is proportional to the square root of the image force, e.g., the spring constant. Based on Langlois’s report, the error in the calculated charge amount is as high as 170%, and may in fact deviate further due to the influence of FIB-milling. In future work, the spring constant of the FIB-milled cantilever should be calibrated using the method of Sader et al. [25], and the quantitativeness as an evaluation system should be verified by evaluating by a combination of particles and substrate that can be used to estimate the contact area. The contact area would be estimated by measuring the surface roughness of the substrate or the adhesion of the particles. Spherical particles would be used to facilitate the estimation of the contact area. The quantitativeness should be verified by comparison with the evaluation result that the entire surface of particles is considered to be charged.

## 4. Conclusions

In the present study, we proposed a novel measurement technique for evaluating the contact electrification characteristics of a single microparticle under controlled contact conditions. The technique can seamlessly transition from a contact test by particle manipulation using nanotweezers to a charge measurement utilizing the AFM cantilever. Moreover, the AFM cantilever was FIB-milled to reduce the spring constant from 0.006 N/m to 0.001 N/m, which increased the sensitivity of the measurement. Finally, the effect of external additives on contact electrification was evaluated using the proposed method.

The throughput of the proposed system is much higher than that of CP-AFM. This is because particle fixing is achieved using nanotweezers in the proposed system, whereas a particle must be glued to the cantilever in CP-AFM. Contact electrification is a phenomenon that appears in various industrial fields. The proposed method has the potential to be applied in a wide variety of areas, such as pharmaceuticals, the food industry, powder coatings, and electrophotography.

## Figures and Tables

**Figure A1:**
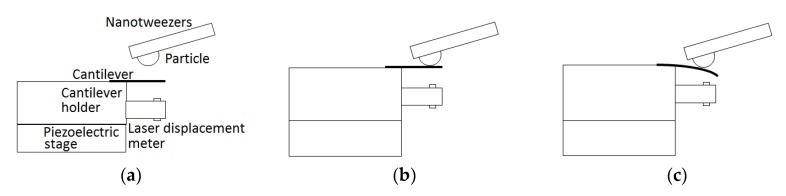
Experimental apparatus for verifying the contact sensing of nanotweezers gripping a particle: (**a**) approach toward particle; (**b**) contact with particle; (**c**) deflection by particle.

**Figure A2:**
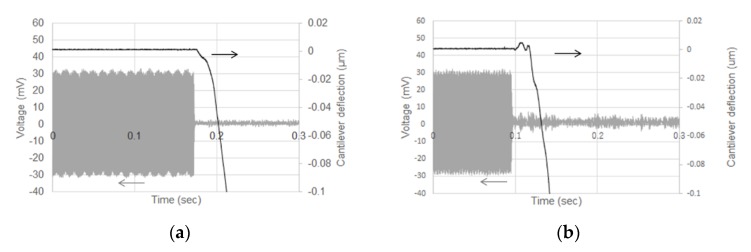
Results obtained simultaneously for the capacitor voltage of the comb drive of the nanotweezers and cantilever deflection: (**a**) without particle; (**b**) with polystyrene particle.

## Appendix A

In the contact test, contact between a particle and the aluminum oxide substrate is determined by the proximity sensing system of the nanotweezers. The probe arms of the nanotweezers can be moved via a comb drive actuator [17], the electrodes of which are connected to a positive feedback oscillation circuit that vibrates the arm mechanically at its resonance frequency. Contact with the substrate is detected based on a change in the oscillation amplitude of the arms.

Although the proximity sensing system is designed to be operated when no particle is being gripped, we have verified that it works even when a particle is being gripped using the image force measurement system of Section 2.3 as follows. As illustrated in Figure A1, a gold-coated cantilever (Olympus OMCL-RC800PB; resonant frequency: 17 kHz, spring constant: 0.11 N/m) was moved upward (velocity: 10 μm/s) by the piezoelectric stage toward the nanotweezers, which gripped a polystyrene particle (diameter: 5 μm). During the approach, the oscillation amplitude (measured as the voltage of the capacitor of the comb drive) and the cantilever deflection were simultaneously acquired with a sampling time of 100 μs. The change in the capacitor voltage and the cantilever deflection as a function of time were compared.

Figure A2 shows the results obtained for the voltage of the capacitor of the comb drive and the cantilever deflection. Both graphs show that the deflection of the cantilever is initially constant and then decreases. The large decrease in the deflection corresponds to bending of the cantilever by either the polystyrene particle or the nanotweezers (when no polystyrene particle is present). In the former case, the initial slight increase in the cantilever deflection is due to the adhesion force between the polystyrene particle and the cantilever, and indicates contact between the two. In the latter case, the start of the decrease in displacement indicates contact between the cantilever and the nanotweezers. In the graphs, the decrease in the amplitude of the voltage coincides with the decrease or initial increase in displacement. Although the voltage fluctuates after contact is made between the particle and cantilever, the overall decrease in the oscillation amplitude is sufficient for detection. The results demonstrate that the contact detection system of the nanotweezers works in the case when a particle is being gripped.
